# A Novel Dual-Function Nitric Oxide Donor Therapy for Preeclampsia—A Proof-of-Principle Study in a Murine Model

**DOI:** 10.3390/antiox12122036

**Published:** 2023-11-23

**Authors:** Diana Pintye, Réka E. Sziva, Lauren A. Biwer, Esilida Sula Karreci, Sonako Jacas, Maxim Mastyugin, Marianna Török, Brett C. Young, Prakash Jagtap, Garry J. Southan, Iris Z. Jaffe, Zsuzsanna K. Zsengellér

**Affiliations:** 1 Department of Medicine, Beth Israel Lahey Health, Boston, MA 02215, USA; dpintye@bidmc.harvard.edu (D.P.); esulakar@bidmc.harvard.edu (E.S.K.); sjacas@bidmc.harvard.edu (S.J.); 2 Department of Obstetrics and Gynecology, Semmelweis University, 1085 Budapest, Hungary; sziva.reka@gmail.com; 3 Molecular Cardiology Research Institute, Tufts Medical Center, Boston, MA 02111, USA; lauren.biwer@yale.edu (L.A.B.); iris.jaffe@tuftsmedicine.org (I.Z.J.); 4 Department of Chemistry, UMass Boston, Boston, MA 02125, USA; maxim.mastyugin001@umb.edu (M.M.); marianna.torok@umb.edu (M.T.); 5 Department of OB/GYN, Beth Israel Lahey Health, Boston, MA 02215, USA; bcyoung@bidmc.harvard.edu; 6 Akkadian Therapeutics, Stoneham, MA 02180, USA; jagtap@akkadiantx.com (P.J.); southan@akkadiantx.com (G.J.S.)

**Keywords:** preeclampsia, nitric oxide, redox modulator, SOD mimetic, sFlt-1, mice

## Abstract

**Background**: Preeclampsia (PE) is a hypertensive disorder of pregnancy that is associated with substantial morbidity and mortality for the mother and fetus. Reduced nitric oxide bioavailability and oxidative stress contribute to the maternal and fetal pathophysiology of PE. In this study, we evaluated the efficacy of a novel dual-function nitric oxide donor/redox modulator, AKT-1005, in reducing PE symptoms in a mouse model of PE. **Method**: The potential therapeutic effect of AKT-1005 was tested in an animal model of Ad.sFlt-1-induced hypertension, proteinuria and glomerular endotheliosis, a model of PE. Pregnant Ad.sFlt-1-overexpressing CD1 mice were randomized into groups administered AKT-1005 (20 mg/kg) or a vehicle using a minipump on gd11 of pregnancy, and the impact on blood pressure and renal and placental damage were assessed. **Results**: In healthy female mice, ex vivo treatment of resistance vessels with AKT-1005 induced vasorelaxation, and 6 days of treatment in vivo did not significantly alter blood pressure with or without pregnancy. When given for 6 days during pregnancy along with Ad.sFlt-1-induced PE, AKT-1005 significantly increased plasma nitrate levels and reduced hypertension, renal endotheliosis and plasma cystatin C. In the placenta, AKT-1005 improved placental function, with reduced oxidative stress and increased endothelial angiogenesis, as measured by CD31 staining. As such, AKT-1005 treatment attenuated the Ad.sFlt-1-induced increase in placental and free plasma soluble endoglin expression. **Conclusions**: These data suggest that AKT-1005 significantly attenuates the sFlt-1-induced PE phenotypes by inhibiting oxidative stress, the anti-angiogenic response, and increasing NO bioavailability. Additional research is warranted to investigate the role of AKT-1005 as a novel therapeutic agent for vascular disorders such as preeclampsia.

## 1. Introduction

Preeclampsia (PE), defined as new-onset hypertension with end organ damage in late pregnancy, is a prevalent disorder of pregnancy that has significant implications for both the pregnant person and the fetus. Preeclampsia affects 5–7% of pregnancies worldwide, leading to 70,000–80,000 maternal and 500,000 perinatal deaths worldwide every year [1,2,3,4]. Even in high-income countries, including the USA, approximately 15% of maternal deaths in the USA can be attributed to hypertensive disorders, with a higher proportion among US-born black women [4]. No therapy to treat the underlying causes is currently available. The most effective recourse is delivery of the pregnancy, which may be necessary at a term or preterm gestational age. Preterm deliveries are a leading cause of neonatal morbidity and mortality. This complication is costly; the cost of PE in the USA alone in 2012 was estimated to be USD 2.18 billion, mostly secondary to premature births [5]. Current care during pregnancy is limited to managing progressive symptoms: controlling hypertension and minimizing the risk of eclampsia (seizures caused by PE) with magnesium sulfate. Therapies that target the underlying causes of PE at an early stage of pregnancy could prevent the life-threatening hypertension and organ dysfunction that characterizes preeclampsia and reset the cycle of redox imbalance/acute injury. The development of therapy to mitigate preeclampsia could have substantial benefits.

While the etiology and pathogenesis of PE remains elusive to date, placental ischemia/hypoxia due to impaired placental development and the subsequent release of anti-angiogenic factors (such as soluble fms-like tyrosine kinase 1 or sF1t-1 and soluble Endoglin or sEng) are early components of the pathophysiology [6,7,8,9,10]. A growing body of evidence reveals that impaired spiral artery remodeling occurs in early PE pregnancies, leading to placental ischemia, resulting in the abnormal secretion of anti-angiogenic factors, including sFlt-1 and sEng [6,7,8]. These anti-angiogenic factors impair trophoblast mitochondrial function, leading to the release of reactive oxygen species (ROS). ROS have been shown to stabilize hypoxia-inducible factor 1 alpha (HIF1A), which, in turn, induces the transcription and release of the anti-angiogenic factors sFlt-1 and sEng from trophoblasts [8]. The ROS and reactive nitrogen species (RNS) cause impaired mitochondrial function in pre-eclamptic placentas, both in humans and in animal models of PE [11,12,13,14,15,16,17]. sFlt-1 is a soluble vascular endothelial growth factor (VEGF) receptor that scavenges the circulating VEGFs necessary for normal endothelial function. In the absence of VEGF, endothelial damage leads to glomerular endotheliosis and proteinuria in the kidney and the vasoconstriction of resistance vessels, which together, are thought to contribute to the development of hypertension. Indeed, in several animal models, the overexpression of sFlt-1 alone during pregnancy is sufficient to produce the phenotype of hypertension, renal glomerular endotheliosis, placental dysfunction and proteinuria. sFlt-1 is increased in human PE and is now an FDA-approved biomarker [18].

Nitric oxide (NO) is thought to have a key role in PE development. NO is a potent vasodilator formed by NO synthases (NOS), including endothelial and inducible NOS (eNOS, iNOS) [19]. NO production increases during normal gestation, contributing to the normal decline in peripheral vascular resistance [20,21,22,23]. eNOS and iNOS are expressed by fetal and placental tissues [21,22], where local NO facilitates spiral artery remodeling. However, iNOS and eNOS activities are decreased in pre-eclamptic placentae [24]. In mice, a lack of eNOS aggravates the sFlt1-induced PE -phenotype. Various studies show that enhancing NO bioavailability has some therapeutic potential in PE [25,26,27,28]; however, the potential benefits of NO delivery are limited by inefficient delivery, the extremely rapid scavenging of NO by ROS, and side effects including headache and hypotension.

AKT-1005 is a novel dual-function NO-donor and redox modulator. AKT-1005 is an organic nitrate NO-donor and a potent redox catalyst by virtue of its nitroxide functional group, which scavenges ROS, including superoxide. We have shown that AKT-1005 can target two components underlying the pathophysiology of PE by raising NO: the imbalance in oxygen-centered and nitrogen-centered reactive species and free-radicals, and maternal hypertension. We recently published data on villous explants stressed by hypoxia and found that AKT-1005 reduced ROS production, reduced the expression of HIF1A transcription factor and improved angiogenic balance as well as mitochondrial function in the trophoblast cells of explant tissues [29]. By eliminating ROS at the site of NO delivery, AKT-1005 delivers NO efficiently, without toxic side products such as peroxynitrite.

As there is currently no effective treatment available that addresses oxidative stress and the early contributors to the development of PE, we set out to test the redox modulator/NO donation effects of AKT-1005 in an in vivo PE model. Here, we demonstrate that AKT-1005 ameliorate the hypertension, improve NO bioavailability, kidney damage and placental dysfunction in a mouse model of sFlt-1-induced preeclampsia.

## 2. Materials and Methods

### 2.1. Materials

AKT-1005 (2,2,5,5-tetramethyl-1-pyrrolidinyloxy-3-yl)-methyl-nitrate was synthesized at Akkadian Therapeutics (Leominster, MA, USA). HPCD (2-Hydroxypropyl)-β-cyclodextrin was purchased from Millipore-Sigma, Danvers, MA, USA.

### 2.2. Vascular Function Studies

Rings from third-order mesenteric resistance arteries were harvested from female CD1 mice and mounted (Danish Myo Technology Aarhus, Hinnerup, Denmark) for isometric tension recordings using PowerLab 35 software (AD Instruments, Dunedin, New Zealand) as previously described. Concentration–response relaxation curves were built in the presence of AKT-1005 or the vehicle by precontracting vessels with phenylephrine at 3 μM. Data are presented as 8 individual mesenteric artery rings from 5 mice/group [30,31].

### 2.3. Animal Model and AKT-1005 Treatment

All animal protocols were approved by the Beth Israel Deaconess Medical Center Institutional Animal Care and Use Committee (Protocol # 002-2023; Approval Date # 15 March 2023). Seven–nine-week-old female CD-1 [Crl:CD1(ICR)] mice were purchased from Charles River Laboratories (Wilmington, MA, USA) and acclimated for at least one week before the start of the experiment. Animals were housed in a temperature- and humidity-regulated environment with a 12 h light cycle. For timed pregnancy experiments, female mice (8 to 12 weeks old) were placed with a stud male for two nights. The observation of a copulation plug the following morning was denoted as gestational day 0 (gd0) and the females were removed from the cage. Pregnant females were injected on gd8 with murine sFlt-1 adenovirus diluted in sterile phosphate-buffered saline (PBS) via the tail vein under isoflurane anesthesia, as described previously [32,33]. The recombinant adenovirus expressing murine sFlt-1 was amplified at a commercial facility (Vector Biolabs, Philadelphia, PA, USA). Within 48 h, plasma mouse sFlt-1 levels were confirmed using a mouse sFlt-1 ELISA kit (R&D Systems, Inc., Minneapolis, MN, USA) and animals were stratified to the treatment or control groups. Both groups were treated with AKT-1005 20 mg/kg (n = 25) or a DMSO/PEG vehicle (n = 22) via minipumps. Alzet osmotic minipumps (Mini-Osmotic Pump; model 2004) were prepared in sterile conditions. AKT-1005 and the vehicle (DMSO/PEG) were prepared according to the manufacturer’s recommendations. Minipumps were loaded using an Alzet loading syringe, and then, implanted sub-dermally in the upper back of the mouse. At termination of experiment, blood, urine and tissue collection were achieved, as previously described [32,33]. Each embryo and corresponding placenta were weighed. Resorption rates were calculated.

### 2.4. Urine Collections

Urine was collected on gd0 and gd17. Individual mice were placed in clean, empty plastic cages, and spot urine samples were collected from the cage floors via pipette. Spot urine samples were centrifuged for 5 min at 5000 rpm. Clarified urine was transferred to a separate 1.5 mL Eppendorf tube and was stored at −80 °C. Cystatin C ELISA was carried out following the manufacturer’s recommendations (R&D Systems, Minneapolis, MN, USA).

### 2.5. Light and Electron Microscopy

Kidney cortex fragments fixed in 10% formalin for 24 h were transferred onto tissue cassettes for paraffin-embedding. Four µm kidney sections were stained with periodic acid–Schiff (PAS) stain. Glomeruli were scored blindly by a pathologist to assess the severity of endotheliosis. Multiple sections and glomeruli per kidney were reviewed, and a score of 0, 0.5, 1 or 1.5 was assigned to each mouse kidney based on the openness of capillary loops and size of glomeruli (swollen or shrunken). A score of 0 was assigned to kidneys with no pathology, 0.5–1 indicated partial obstruction and 1.5 indicated severe evidence of glomerular endotheliosis.

For electron microscopy, kidneys were immediately removed after termination and fixed in 2% formaldehyde and 2.5% glutaraldehyde in 0.1 M sodium cacodylate buffer, pH 7.4; dehydrated; and embedded in an araldite-EM bed 812 mixture. Large sections were cut perpendicular to the renal capsule, containing cortex and medulla. One-micron sections, stained with 1% methylene blue, were analyzed in a blinded fashion for morphologic alterations, as described previously [33,34]. Multiple ultrathin sections were then cut from each sample and examined using a JEOL 1011 Transmission Electron Microscope, with a Hamamatsu Orca-HR Digital Camera and an Advanced Microscopy Techniques (AMT) Corp. image capture system to acquire glomerular images.

For placental histology, 4 placentas per mouse were isolated immediately after sacrifice. One fragment was placed in a 4% paraformaldehyde solution for subsequent optical microscopy analysis. Staining was first performed with H&E for morphology assessment. To quantify the vasculature area within the labyrinth, we used immunohistochemistry to stain for CD34 (rabbit monoclonal anti-CD34, 1:500, Abcam, ab81289) with hematoxylin counterstaining. Morphometric measurements were performed using Fiji software version 2 (National Institute of Health (NIH), Bethesda, MD, USA; https://imagej.net/software/fiji/ (accessed on 12 November 2023)) with 20× objective magnification.

### 2.6. Blood Pressure Measurement

Systolic blood pressure (SBP), diastolic blood pressure (DBP) and mean arterial pressure (MAP) were measured from the mouse tail using a CODA non-invasive plethysmography blood pressure transducer (Kent Scientific Corporation, Torrington, CT, USA) at baseline, gd11, gd14, gd16 and gd17 [35].

### 2.7. Enzyme-Linked Immunosorbent Assay (ELISA)

Soluble Flt-1 (sFlt-1) in plasma was measured by ELISA using a mouse VEGF receptor 1 (VEGFR1) Quantikine kit (Cat# MVR100, R & D Systems, Minneapolis, MN, USA) following the manufacturer’s instructions. This assay has an intra-assay coefficient of variation of 2.6–3.8% and an inter-assay coefficient of variation of 5.5–9.8% [11].

### 2.8. Statistical Analysis

Longitudinal data (hemodynamic data, serial sFlt-1 measures) were compared using 2-way repeated-measures analysis of variance (ANOVA), followed by Šidák’s or Bonferroni’s post hoc test, as applicable. Data with multiple measurements per n were compared using 2-way ANOVA. Data comparing 3 or more groups were analyzed using 1-way ANOVA with Tukey’s multiple comparisons test or Kruskal–Wallis with Dunn’s post-test, depending on data-distribution. Two-group comparisons were made using either paired or unpaired 2-tailed *t* tests with or without Welch’s correction, as applicable. GraphPad Prism version 10.06 was used for all analyses. A *p* value of less than 0.05 was considered statistically significant (*p* < 0.05).

## 3. Results

### 3.1. AKT-1005 Has Vasodilating Effect on Resistance Vessels Ex Vivo and Does Not Cause Hypotension in Control Mice

**Figure 1:**
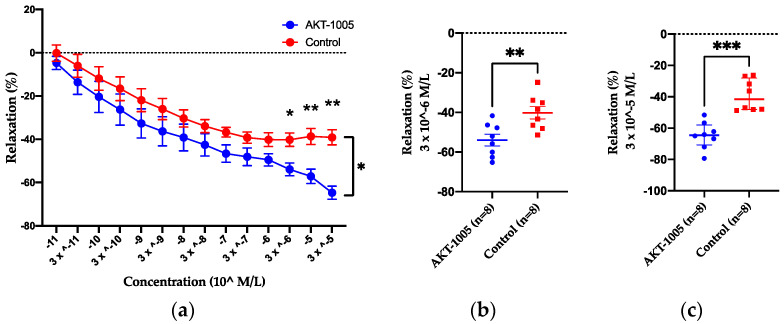
**AKT-1005 treatment caused vascular relaxation in third-order mesenteric arteries of CD1 non-pregnant control mice.** (**a**) Dose–response curve of third-order mesenteric arteries from CD1 non-pregnant mice were used for wire-myography studies. Percentage of vasorelaxation shown on phenylephrine contracted vessels, as analyzed in intact arterial rings. n = 8 vessels from 5 mice/group. Mean ± SEM. Repeated-measures ANOVA: *: *p* < 0.05, **: *p* < 0.01, ***: *p* < 0.001. % vasorelaxation with (**b**,**c**) 3 × 10^−6^ M/L and (**c**) 3 × 10^−5^ M/L AKT-1005 is shown on phenylephrine-contracted vessels. n = 8 vessels from 5 mice/group. Mean ± SEM. Unpaired *t*-test: **: *p* < 0.01, ***: *p* < 0.001.

Our data indicated that AKT-1005 is a ROS scavenger and increases NO [29]. Consequently, we tested whether it can directly dilate resistance arteries. We evaluated the vasodilatory properties of AKT-1005 in mesenteric arteries from CD1 non-pregnant mice. After precontraction of the vessels with phenylephrine, AKT-1005 induced moderate vasorelaxation at concentrations of 3 × 10^−6^ (*p* < 0.05) and at higher concentrations compared to vehicle treatment (Figure 1a–c). In addition, we investigated the effect of AKT-1005 on blood pressure in normotensive mice (non-pregnant and pregnant) and we found no changes after 6 days of treatment (Supplemental Figure S1), suggesting that it has similar action to other organic nitrite NO donors and it does not cause systemic hypotension in healthy mice.

### 3.2. AKT-1005 Increases NO Bioavailability and Ameliorates Hypertension in Ad.sFlt-1-Transfected Mice

**Figure 2:**
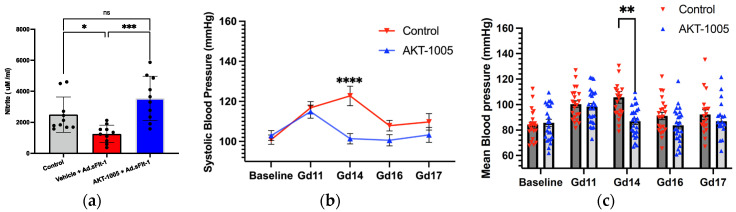
**AKT-1005 treatment reduced systolic BP and mean arterial blood pressure (MAP) in Ad.sFlt-1-injected CD1 pregnant mice and improved plasma NO availability.** (**a**) Pregnant mice given Ad.sFlt-1 on gd8 (induction of disease) and administered either AKT-1005 (n = 10) (20 mg/kg/day) or vehicle (n = 10), starting on gd11 up to gd17. Plasma nitrite concentrations were reduced (Gd17) in Ad.sFlt-1-injected mice and were restored after AKT-1005 treatment. Baseline measurements were taken before treatment with Ad.sFlt-1 in pregnant mice. One-way repeated-measures ANOVA—Gd17: ***: *p* < 0.001, and *: *p* < 0.05. (**b**,**c**) Pregnant mice were treated as in (**a**). Systolic BP and MAP are significantly lower in the AKT-1005-treated group (n = 26) compared to the vehicle (n = 22). Two-way repeated-measures ANOVA—Gd14: ****: *p* < 0.0001, and **: *p* < 0.01, ns: not significant.

Next, we evaluated the in vivo effects of AKT-1005 in the sFlt-1-induced mouse model of PE. AKT-1005 was administered at 20 mg/kg/day via minipump to sFlt-1-overexpressing CD1 pregnant mice. Minipump administration was chosen since pharmacokinetic (PK) analysis of AKT-1005 has indicated a short half-life via all dosing routes (Supplemental Figure S2). The plasma levels of AKT-1005 were 40–160 ng/mL (0.2–0.8 μM) at gd17 after six days of administration via minipump (Supplemental Figure S3). The plasma levels of the NO metabolite, nitrite, were measured at gd17 and demonstrated that nitrite levels are significantly reduced in Ad.sFlt-1-overexpressing mice, indicative of reduced NO bioavailability. AKT-1005 treatment significantly increased plasma nitrite levels during pregnancy, restoring the level back to the level of non-sFlt1-treated pregnant mice (Figure 2a).

The peak systolic blood pressure during sFlt1-induced PE was significantly lower in the AKT-1005-treated group at gd14 (109 mmHg); compared to the control vehicle-treated group (122.7 mmHg) (*p* < 0.0001; Figure 2b,c). The mean arterial pressure (MAP) in AKT-1005-treated animals was not different from baseline at all timepoints. In addition, plasma sFlt-1 concentrations were elevated to the same extent in both the AKT-1005-treated (87,103 ± 8993 pg/mL, n = 9) and vehicle-treated mice (75,631 ± 13,448 pg/mL, n = 9) at gd17, which indicates that the study drug acted downstream of the PE inducer in this model.

### 3.3. AKT-1005 Prevents Renal Glomerular Endotheliosis and Improves Kidney Function in Ad.sFlt-1-Transfected Mice

**Figure 3:**
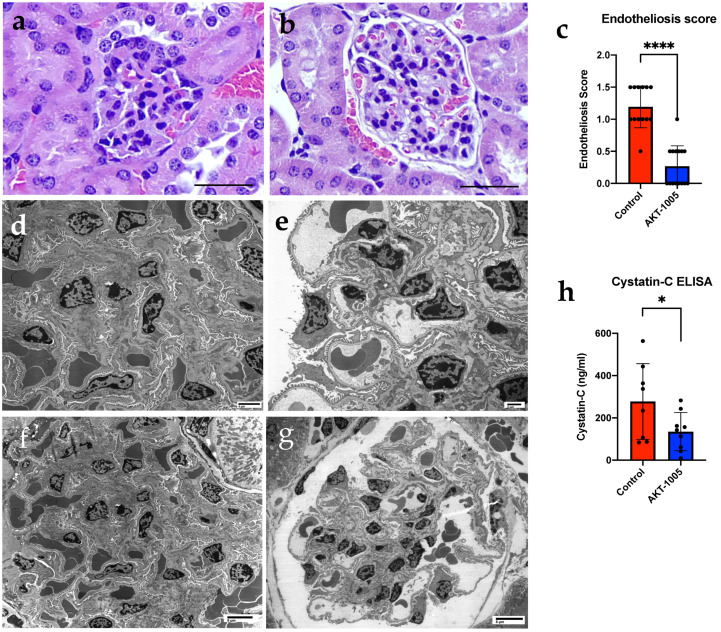
**Kidney morphology and function were improved with AKT-1005 treatment in CD1 mice injected with Ad.sFlt-1.** (**a**,**b**) H&E analysis of kidney tissue from AKT-1005-treated mice shows ameliorated glomerular endotheliosis (**b**) compared to vehicle-treated control mice (**a**) on gd17. Endotheliosis presents as endothelial swelling in the glomeruli of maternal kidneys from sFlt1-overexpressing mice. (**c**) Average glomerular endotheliosis severity scored based on blinded analysis. Mann–Whitney-U test, median (IQR): ****: *p* < 0.0001. n = 6 animals from each group. (**d**–**g**) Electron microscopy analysis of kidney tissue from AKT-1005-treated mice (**e**,**g**) shows ameliorated glomerular endotheliosis compared to vehicle-treated mice (**d**,**f**) (scale bars: (**a**,**b**): 100 μm; (**d**): 4 μm; (**e**): 2 μm; (**f**): 6 μm; and (**g**): 8 μm). (**h**) Cystatin C ELISA from urine samples indicates improved kidney function in AKT-1005-treated mice (n = 9) compared to vehicle control (n = 8). Mann–Whitney-U test, median (IQR): *: *p* < 0.05.

Similar to our previously published data [33], animals overexpressing sFlt-1 demonstrated marked glomerular endotheliosis with occlusion of the capillary lumen (Figure 3a). In contrast, kidneys from AKT-1005-treated animals had more open capillaries and less glomerular damage, with the absence of proteinaceous deposits and distinct mesangium (Figure 3b,c). Electron microscopy confirmed that glomerular endotheliosis was ameliorated in AKT-1005-treated animals (Figure 3e,g) compared to vehicle-treated mice (Figure 3d,f). The cystatin C ELISA indicated that kidney function was improved in the AKT-1005 group compared to vehicle-treated group (*p* < 0.05, Figure 3f).

### 3.4. AKT-1005 Relieves Placental Stress in Ad.sFlt-1-Transfected Mice

**Figure 4:**
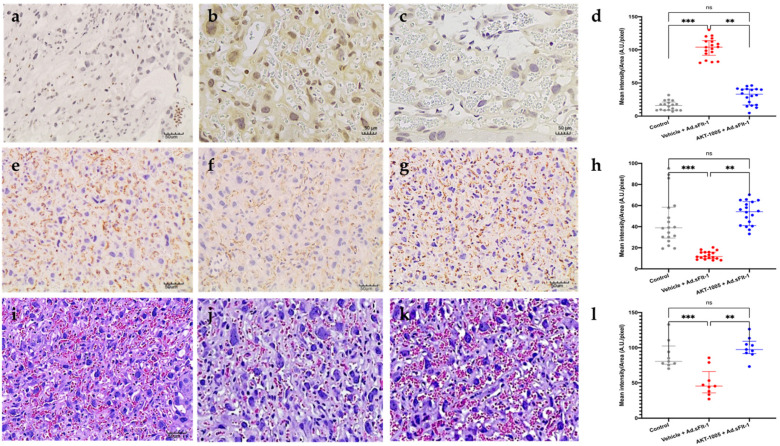
**Effect of AKT-1005 on placental stress in CD1 mice overexpressing sFlt-1.** (**a**–**d**) Nitrotyrosine staining in placenta of pregnant mice given Ad.sFlt-1 on gd8 (induction of disease) and administered either AKT-1005 (20 mg/kg/day, (**c**)) or vehicle (**b**), starting on gd11 up to gd17 ((**a**), control pregnancy). Nitrotyrosine staining (indicative of nitrosative stress and peroxynitrite production) is significantly lower in the AKT-1005-treated group (**c**) compared with vehicle (**b**). (**d**) Quantitation of nitrotyrosine immunostaining: mean optical density was calculated in 6 high-power fields per sample (n = 3 per group). Kruskal–Wallis test, Dunn’s post hoc test, median (IQR). Control vs. Vehicle + Ad.sFlt-1: ***: *p* < 0.001, vehicle + Ad.sFlt-1 vs. AKT-1005 + Ad.sFlt-1: **: *p* < 0.01. (**e**–**h**) At gd17, AKT-1005 treatment improves CD31 staining abundance in the placenta (**g**) compared to vehicle-treated mice (**f**) ((**e**), control pregnancy). (**h**) Quantitation of CD31 immunostaining: mean optical density was calculated in 6 high-power fields per sample (n = 3 per group). Kruskal–Wallis test, Dunn’s post hoc test, median (IQR), control vs. vehicle + Ad.sFlt-1: ***: *p* < 0.001, vehicle + Ad.sFlt-1 vs. AKT-1005 + Ad.sFlt-1: **: *p* < 0.01. (**i**–**l**) At gd17, labyrinthine vasculature appeared collapsed in saline-treated mice (**j**). AKT-1005 improves placental vasculature in mice overexpressing sFlt1 (**k**). ((**i**), control pregnancy). (Original magnification: 20x, bar = 50 μm). (**l**) Quantitation of placental tissue vascular space: mean optical density was calculated in 3 high-power fields per sample (n = 3 per group). Kruskal–Wallis test, Dunn’s post hoc test, median (IQR), control vs. vehicle + Ad.sFlt-1: ***: *p* < 0.001, vehicle + Ad.sFlt-1 vs. AKT-1005 + Ad.sFlt-1: **: *p* < 0.01.

To evaluate the effect of AKT-1005 on the placenta in CD1 mice overexpressing sFlt-1, nitrotyrosine (NT) staining was performed. NT staining (a marker of nitrosative stress and peroxynitrite production) was significantly greater in the placentae of pregnant mice given Ad.sFlt-1 with the vehicle compared to no Ad.sFlt-1 (Figure 4a–d), confirming the induction of placental nitrosative stress by sFlt1 overexpression during pregnancy. AKT-1005-treated mice had less placental NT reactivity (Figure 4c). AKT-1005 treatment also restored placental CD31 staining, a measure of angiogenesis compared to vehicle-treated mice (Figure 4e–h). At gd17, the labyrinthine vasculature appeared collapsed in vehicle-treated mice, and AKT-1005 improved the placental vasculature in mice overexpressing sFlt1 (Figure 4i–l).

### 3.5. AKT-1005 Reduces Anti-Angiogenic Response in Ad.sFlt-1-Transfected Mice

**Figure 5:**
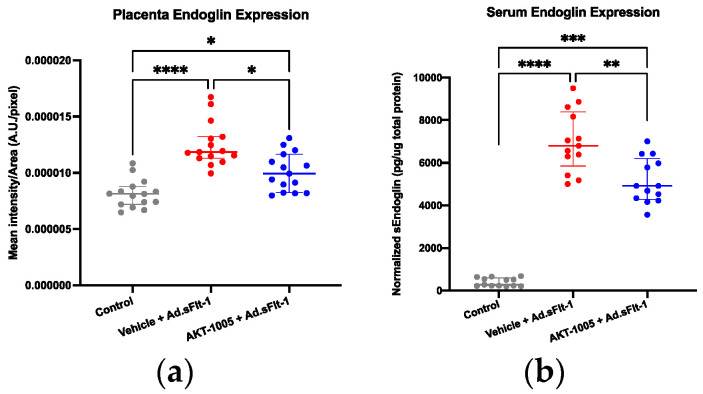
**AKT-1005 treatment reduced both placental and systemic expression of sEndoglin protein (anti-angiogenic response) in Ad.sFlt-1-injected CD1 pregnant mice.** (**a**) Quantitation of placental sEng expression: mean optical density was calculated in 5 high-power fields per sample (n = 3 per group). (**b**) Serum sEng expression was normalized to total protein concentrations in the plasma (n = 13). Kruskal-Wallis test, Dunn’s post-hoc test, Median [IQR], Control vs. Vehicle + Ad.sFlt-1: ****: *p* < 0.0001, Vehicle + Ad.sFlt-1 vs. AKT-1005 + Ad.sFlt-1: *: *p* < 0.1, **: *p* < 0.01, Control vs. AKT-1005 + Ad.sFlt-1: ***: *p* < 0.001.

In response to ischemia, the placenta produces sEng; thus, we measured its levels in the placental tissue and serum. The overexpression of sFlt1 significantly increased placental (Figure 5a) and circulating sEng (Figure 5b). AKT-1005 treatment reduced both the placental (where endoglin is synthesized, Figure 5a) and systemic expression (Figure 5b) of sEng protein in Ad.sFlt-1-injected CD1 pregnant mice.

### 3.6. AKT-1005 Does Not Alter Pregnancy Outcomes in Ad.sFlt-1-Transfected Mice

**Figure 6:**
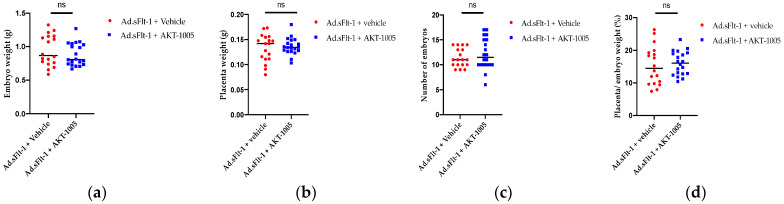
**Pregnancy outcomes were not altered by AKT-1005 treatment.** Mice were given Ad.sFlt-1 at gd8, and then, treated with 20 mg/kg/day of AKT-1005 via minipump from gd11. Tissue was collected at gd17. (**a**) Embryo weight, (**b**) placenta weight, (**c**) number of embryos and (**d**) placental/embryo weights were measured. No significant difference was found between AKT-1005- and vehicle- treated groups at gd17. All data are mean ± SEM, analyzed via unpaired 2-tailed *t* test with Welch’s correction, except H. Unless specified, n = 18 control and n = 20 AKT-1005-treated mice.

We also assessed pregnancy outcomes such as placental and embryo weight (Figure 6a,b), the number of embryos (Figure 6c) and resorption rate (Figure 6d). We found that AKT-1005 did not have any adverse effects.

## 4. Discussion

In this study, we tested a novel dual-action NO donor and redox catalyst, AKT-1005, in a murine model of sFlt-1-induced PE. Our major findings are that in our model of PE, AKT-1005 restored normal blood pressure, reduced nitrosative stress, improved kidney function, decreased placental ROS, improved placental angiogenesis and reduced anti-angiogenic responses.

The current therapies for PE are limited. Besides the delivery of the fetus and placenta, the management of hypertension is a mainstay treatment for PE, but this will not prevent the ongoing progression of the underlying PE disease process, and anti-hypertensive agents are limited in pregnancy. Accordingly, the NHBPEP (National High Blood Pressure Education Program) and ACOG (American College of Obstetricians and Gynecologists) offer recommendations (methyldopa, nifedipine, hydralazine and labetalol as first-line agents), while angiotensin-converting-enzyme (ACE) inhibitors are not recommended during pregnancy [36,37,38]. Organic nitrates such as sublingual nitroglycerine and isosorbide mononitrate have been used in small studies of at-risk pregnancies [25,26], but headaches and the development of tolerance limits its use. Sodium nitroprusside is reserved for extreme emergencies, and magnesium sulfate is only used for seizure prophylaxis in the setting of PE with severe features. However, such treatments do not address the profound sensitivity to vasopressors, such as angiotensin II, and nitric oxide (NO) deficiency. The enhanced vasoconstrictor sensitivity, as well as elevations in sFlt-1, precede the clinical signs and symptoms of PE. Indeed, the overexpression of sFlt-1 in pregnant mice induces angiotensin II sensitivity and hypertension by reducing endothelial NO flux via impairing the phosphorylation of eNOS and promoting oxidative stress in the vasculature [32].

The innovative nature of AKT-1005 is that it targets two broad and inter-related components underlying the pathophysiology of PE—the imbalance in oxygen-centered and nitrogen-centered reactive species and free-radicals, and maternal hypertension. NO-donors would be expected to reduce blood pressure in our PE model via the NO-mediated relaxation of vascular smooth muscle. However, NO reacts rapidly with O2^−^ (as well as other ROS) to form the toxic agent peroxynitrite (ONOO^−^). Thus, the release of NO into an environment rich in O2^−^ can lead to apparently paradoxical tissue damage mediated by ONOO^−^. AKT-1005 is not only an efficient NO-donor, but is also a potent redox catalyst that deactivates ROS [29], and in particular, superoxide (O2^−^), in the vicinity of NO release, thereby avoiding the unwanted formation of ONOO^−^. A further consequence of this is that the flux of bioavailable NO from AKT-1005 may be expected to be higher than for other NO donors because there are less ROS to scavenge the released NO. Indeed, AKT-1005 significantly suppressed the formation of 3-nitrotyrosine (3-NT), a marker for ONOO^−^ and nitrosative stress, in our animal models of PE and reduced placental nitrotyrosine.

It is also a potential benefit that during the AKT-1005 treatment period, the maternal blood pressure was restored to the baseline values, in contrast to the vehicle-treated animals. There is no evidence of hypotension, even when AKT-1005 is given to normal, non-hypertensive pregnant and non-pregnant mice under the same protocol (Supplementary Data). Moreover, wire myography studies in control mesenteric vessels showed the vasodilating capacity of AKT-1005. This may be attributable to the nature of organic nitrite NO-donors (as opposed to direct acting NO-donors) or to the modest concentrations of AKT-1005 observed in the circulation. This is a major benefit as potential hypotension is a concern, limiting the first-line use of many antihypertensive agents in PE.

AKT-1005 and its non-nitrate analog, HMP, have been shown to be efficient deactivators of superoxide and peroxyl radicals in vitro [29,39]. This is due to their common structural feature: both contain the nitroxide function that can react repeatedly with multiple molecules of superoxide, behaving as SOD mimetic catalysts. The role of the anti-ROS actions of AKT-1005 may be two-fold. Firstly, it prevents the side reactions of NO with ROS and allows for local delivery of NO that is free of reactive nitrogen species (RNS). Secondly, it corrects the redox imbalance that has been shown to occur in PE tissues and that may be instrumental in the development of PE. This later point is supported in the current study, in which placental oxidative stress was reduced by AKT-1005. Most likely, this effect and the increased NO bioavailability are responsible for the downstream effect on placental vascular function, which was evidenced by increased CD31 staining and improved placental vascular morphology.

In this context, it is pertinent that AKT-1005 treatment also resulted in a reduced anti-angiogenic response, which has been shown by us previously in a placental explant model induced by hypoxic stress [29]. In that study, we showed that AKT-1005 reduced both the oxidative stress and the upregulation of hypoxia-inducible factor alpha (HIF1A). HIF1A is a transcription factor for sFlt-1 and sEng. In the present study, we attempted to measure HIF1A in various tissues of our mice, but we did not see significant changes with treatment, which could be explained by the rapid degradation of this factor in vivo.

Pregnancy outcomes were not altered after AKT-1005 treatment, which implies that the treatment did not cause adverse effects.

Kidney endotheliosis occurs in human PE and is reproduced in the sFlt1-induced mouse model. AKT-1005 improved renal endotheliosis, potentially due to the reduced blood pressure and potential vascular and glomerular protective effects of the drug. We also show that cystatin C, a non-specific marker of kidney dysfunction, is reduced by treatment with AKT-1005, emphasizing that not only systemic but also organ-specific protection is achieved by AKT-1005. It is well-documented that women with preexisting kidney disease or hypertension are most likely to develop PE during their pregnancy. Dupont et al., have reported that uninephrectomized mice develop PE with high blood pressure via a mechanism involving kynurenine and tryptophan [35]. Therefore, the kidney’s involvement in PE is an important factor, and the study drug’s preservation of organ function is notable.

It’s also noteworthy that many women with PE will remain hypertensive postnatally. Studies from our group have identified that the activation of smooth muscle cell mineralocorticoid receptors contributes to postpartum hypertension in response to common stresses [40]. Future studies may explore the efficacy of AKT-1005 in reducing hypertension postpartum.

## 5. Conclusions

Our findings suggest that AKT-1005 significantly attenuates the sFlt-1-induced PE phenotypes with decreased oxidative stress and anti-angiogenic response, reduced nitrosative stress and increased NO bioavailability. This results in a resolution of hypertension, renal dysfunction, and placental ischemia. In non-preeclamptic mice, AKT-1005 is a modest vasodilator that does not lower blood pressure. These data provide the first proof-of-concept data supporting that AKT-1005 could be beneficial during PE, using a clinically relevant mouse model of PE. While AKT-1005 may provide an exciting lead compound, it may well be that certain attributes of AKT-1005 can be modified to improve its pharmacologic properties (solubility, stability and pharmaco-kinetic properties). This optimization of a lead compound, AKT-1005, will generate an improved version that can be tuned to intravenous delivery for clinical settings or, possibly, oral therapy for non-clinical settings. Further studies are needed to assess the safety and efficacy of this type of redox-modulator therapy in other animal models of PE. This would be an important next step in the clinical development of novel therapies to improve maternal and fetal outcomes in PE.

## Data Availability

All data can be accessed with the approval of Akkadian Therapeutics.

## Supplementary Materials

The following supporting information can be downloaded at: https://www.mdpi.com/article/10.3390/antiox12122036/s1, Figure S1: AKT-1005 does not alter blood pressure in normotensive pregnant or non-pregnant mice; Figure S2. Pharmacokinetic (PK) analysis of AKT-1005 (20 mg/kg) in non-pregnant female CD1 mice; Figure S3: Plasma levels of AKT-1005 were detected by Mass Spectrometry after 6 days of minipump treatment @20 mg/kg/day in pregnant CD1 mice (n = 6). Treatment started on gd11 and terminated at gd17. M48-CTL received vehicle instead of AKT—1005; Table S1: PK analysis and definitions.
