# Supplementary material for: A Novel Dual-Function Nitric Oxide Donor Therapy for Preeclampsia—A Proof-of-Principle Study in a Murine Model
Source: Antioxidants (Basel). 2023 Nov 23;12(12):2036. doi: 10.3390/antiox12122036 (PMC10741224; doi:10.3390/antiox12122036)
Supplement: Supplementary file 1 [file antioxidants-12-02036-s001.zip › antioxidants-2701240-supplementary.pdf]

Supplemental Figure S1.

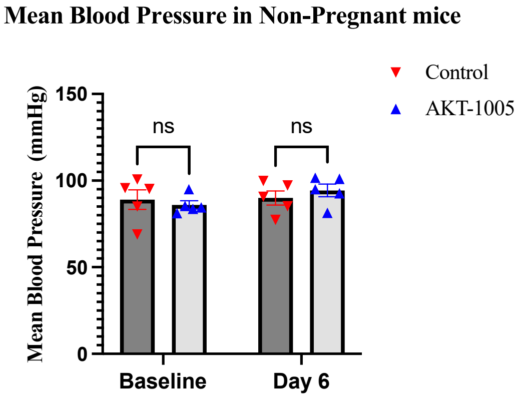

(a)

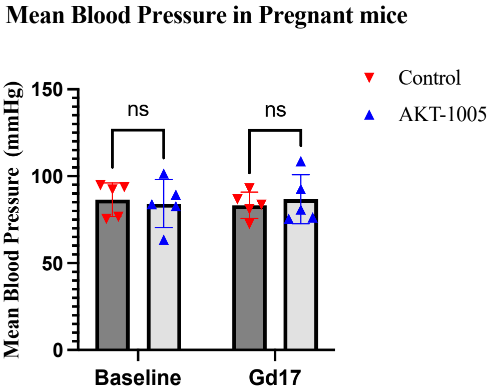

(b)

Figure S1. AKT-1005 does not alter blood pressure in normotensive pregnant or non-pregnant mice. Mice were treated with AKT-1005 via minipumps @20 mg/kg dose for 6 days and mean blood pressure was measured at baseline and at day 6 in non-pregnant mice (SuppF1a) and at gd 0 and Gd17 in pregnant mice (SuppF1b) (Two-way repeated-measures ANOVA. Mean ± SEM, n=6, ns=non-significant.)

Supplemental Figure S2.

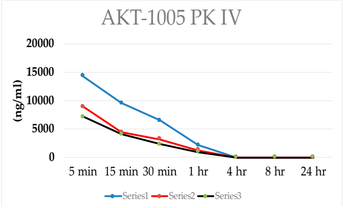

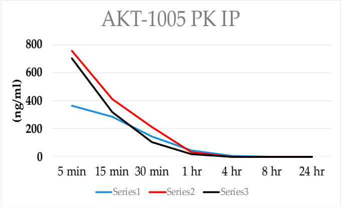

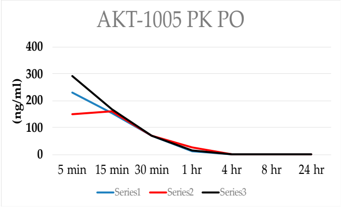

(a) (b) (c)

Figure S2. Pharmacokinetic (PK) analysis of AKT-1005 (20 mg/kg) in non-pregnant female CD1 mice (n=3). x-axis: time, y-axis: concentration of AKT-1005 (ng/ml). (a) Intravenous/IV, (b) intraperitoneal/IP and (c) per os/PO administration.

Supplemental Table S1.

Parameter	IV	IP	PO	Units
Tmax	0.083	0.25	0.25	hrs
Cmax	10086.667	605.67	224.67	ng/ mL
Tlast	1	4	2	hrs
Clast	1463.333	2.51	18.7	ng/ mL
AUClast	3804.519	330.568	142.621	ng/ mL*hr
AUMClast	1237.892	206.97	76.413	ng/ mL*hr
MRTlast	0.325	0.626	0.536	hrs
Thalf	0.318	0.583	0.674	hrs
Tinf	4.571	4.547	4.032	hrs

Table S1. PK analysis and definitions:

1. Cmax, Tmax and Cmax: Are the value and the time of maximum observed concentration, respectively. If the maximum concentration is not unique, the first maximum is used. For steady state data, the maximum value between the dosing intervals is considered. Cmax is the dose normalized maximum observed concentration.
2. Clast and Tlast: Are the last measurable positive concentration and the corresponding time, respectively.
3. AUClast: Is the area under the concentration vs. time curve from the first observed to last measurable concentration.
4. AUMClast: Is the first moment of the concentration vs. time curve from the first observed to last measurable concentration.
5. MRTlast: Is the mean residence time from the first observed to last measurable concentration.

Supplemental Figure S3.

Sample Name	Timepoint	Length of treatment	TotConc AKT-1005 (ng/ml)
M42	Gd17	6 days	160
M45	Gd17	6 days	60
M46	Gd17	6 days	80
M48-CTL	Gd17	6 days	0
M54	Gd17	6 days	40
M58	Gd17	6 days	120
M61	Gd17	6 days	70

Figure S3. Plasma levels of AKT-1005 were detected by Mass Spectrometry after 6 days of minipump treatment @20mg/kg/day in pregnant CD1 mice (n=6). Treatment started on gd11 and terminated at gd17. M48-CTL received vehicle instead of AKT--1005.
